# Pulmonary metastasis as sole manifestation of relapse in previously treated localised prostate cancer: three exceptional case reports

**DOI:** 10.3332/ecancer.2016.645

**Published:** 2016-06-07

**Authors:** Joaquim Peres Gago, Gabriela Câmara, Jorge Dionísio, Ana Opinião

**Affiliations:** 1 Department of Medical Oncology, Instituto Português de Oncologia de Lisboa Francisco Gentil, Lisbon 1099-023, Portugal; 2 Department of Pulmonology, Instituto Português de Oncologia de Lisboa Francisco Gentil, Lisbon 1099-023, Portugal

**Keywords:** prostate cancer, adenocarcinoma, solitary pulmonary metastasis

## Abstract

Metastatic prostate cancer recurrence after definitive local therapy can occur in any tissue. Usually, the first affected site is the bone. Lung metastases without bone or lymph node involvement are extremely rare in patients with prostate cancer, and only a handful of cases are reported in the literature. In several other malignancies, such as breast cancer, sarcomas, colorectal cancer, and renal cell carcinoma, long-term disease-free survival has been reported after resection of solitary pulmonary metastases.

We present three unusual cases of isolated pulmonary recurrence of prostate cancer after initial definitive local therapy. One of the patients underwent resection of the lung metastasis, resulting in a long-term disease-free survival.

Both surgical excision of solitary and oligometastatic lung secondary lesions and systemic therapy can play an important role in long-term disease control. Surgery should be considered for selected and well-informed patients with pulmonary metastasis after primary localised treatment for prostate cancer.

## Introduction

Metastatic prostate cancer recurrence after definitive local therapy can occur in any tissue. The most common metastatic site is bone. Post-mortem studies found evidence of metastases to the lung in more than 40% of men with prostate cancer (PCa) [1, 2]. However, lung metastases without bone or lymph node involvement are extremely rare and only a handful of cases are reported in the literature [3–8].

In several other malignancies, such as breast cancer, sarcomas, colorectal cancer, and renal cell carcinoma, long-term disease-free survival has been reported after resection of solitary pulmonary metastasis. Therefore, in the context of lung oligometastatic disease, the question arises as to whether selected patients with solitary pulmonary metastasis of PCa could indeed benefit from the resection of these lesions. Very limited data from other published case reports already showed some potential benefit [9–11]. Also, very often hormone therapy shows favourable and long-term clinical responses in this context.

We present three unusual cases of isolated pulmonary recurrence of PCa after initial definitive local therapy.

## Case report 1

The first case refers to a 63-year-old, Caucasian, non-smoker male patient, with a personal history of dyslipidaemia and grade III haemorrhoids. He was referred to our institute after radical prostatectomy and bilateral lymphadenectomy. Pathological examination of the resection specimen revealed a Gleason 9 prostate adenocarcinoma, pT_3b_ pN_o_ with both perineural and vascular invasion, extra-capsular extension, and positive surgical margins. The pre- and post-operative PSA level was 25.4 and 12.5 ng/ml, respectively.

He underwent adjuvant external beam radiotherapy (EBRT) and adjuvant hormonal-therapy (HT) with PSA normalisation (0.17 ng/ml).

**Figure 1.**
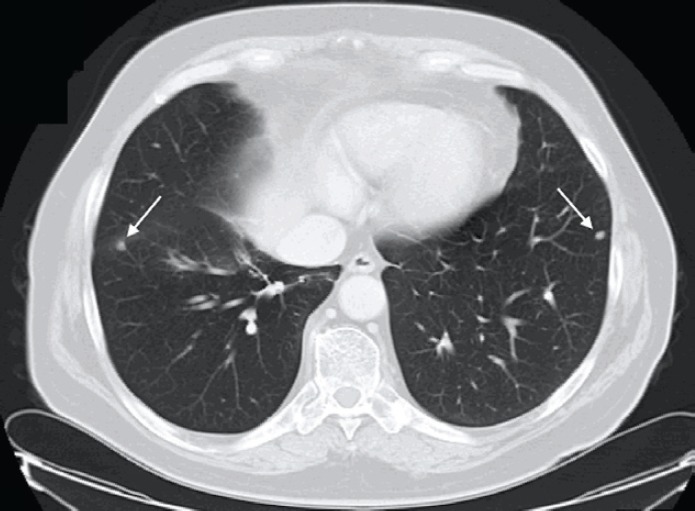
Patient #1. Thoracic CT scan documenting micronodules in the right and left lungs (lower lobes). Arrows indicate secondary lesions.

Approximately, five years after surgery, due to an increase in PSA level (4.3 ng/ml), an abdominal and pelvic CT scan was performed. On the lower thoracic images, non-specific micronodules in the lower lobe of his right lung were documented (Figure 1).

Bronchoscopy was unremarkable, and the samples collected were negative for neoplastic cells. Positron emission tomography–computed tomography–flurodeoxyglucose (PET–CT–FDG) did not detect right lung or infracarinal topography hypermetabolism [only a focally increased standardized uptake value (SUV) in the root of the left pulmonary hilum, suggesting left lung inflammation]. It was decided to proceed with CT scan surveillance.

Prostate-specific antigen (PSA) levels had a progressive increase during the following year and reached 12.3 ng/ml one year later. Bronchoscopy was then repeated, and bronchial secretions/mucus, and endobronchial ultrasound (EBUS) of a subcarinal node confirmed the presence of metastases of carcinoma. Immunohistochemical profile consistent with prostate carcinoma (PSA immunoexpression). Bone scan discarded bone metastases.

After progressing to castration-resistant PCa, he was enrolled in a randomised clinical trial of aflibercept versus placebo in combination with docetaxel 75 mg/m^2^ plus prednisolone 5 mg bid. After six cycles, docetaxel was halted due to toxicity (neurological, ophthalmologic – tearing, persistent cough, and anorexia). He completed two more cycles of experimental treatment alone, but the treatment was discontinued due to persistent toxicity. Imaging re-evaluation at that time demonstrated a complete response.

Eight months after stopping the treatment, PSA levels started to rise (reaching 10.86 ng/dL) without clinical or radiological evidence of disease.

Three months later, thoracic CT scan was repeated and documented a significant increase in the subcarinal lymph node dimensions. Treatment with docetaxel and prednisolone was resumed but was discontinued 4 months later as a result of severe toxicity, which motivated two hospital admissions for septic shock (both with respiratory origin).

The patient went through two additional lines of chemotherapy, due to disease progression (included first the development of superior vena cava syndrome and later of forearm cutaneous metastases). He died two years after the diagnosis of metastatic PCa, on account of a healthcare-related pneumonia.

## Case report 2

We report a 62-year-old, Caucasian male patient. He was a retired electrician with a personal history of hypertension and dyslipidaemia, non-smoker. The patient had undergone a radical prostatectomy 7 years before. The surgical specimen showed a pT_3a_ pN_x_ Gleason 7 prostate adenocarcinoma, with perineural invasion and extracapsular extension; surgical margins were negative. Pre- and post-operative PSA was 4.5 and 0.72 ng/ml, respectively.

Two years after surgery, he developed biochemical failure. Bone scan showed only degenerative bone disease and was otherwise normal. There were no signs of local recurrence on pelvic CT scan. He was treated with salvage EBRT and maintained serum PSA levels around 2 ng/mL after radiotherapy.

Twenty-two months after completion of radiation therapy, the patient developed a lower left lobe pneumonia. As an inpatient, he did a thoracic CT scan that detected small nodules in the same location as the infectious process (lower lobe of the left lung). He repeated this examination 1 month after resolution of his pneumonia, showing dimensional increase in all the lung lesions.

**Figure 2.**
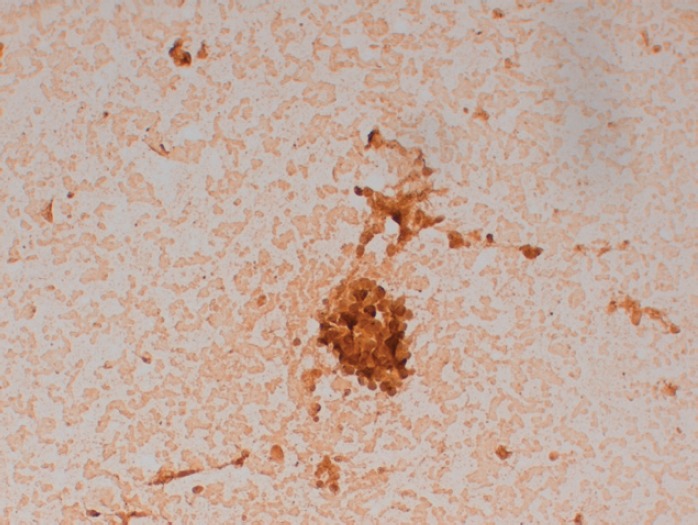
Lung wedge resection of patient #2 showing positive staining for PSA. Original magnification x200.

He was then submitted to a wedge resection of the left lung nodules. Surgical specimen contained a malignant neoplasm of glandular pattern (CK7, CK20 negative, and PSA positive), consistent with PCa (Figure 2).

After surgery, PSA levels decreased to 5.14 ng/ml, and he began treatment with a luteinising hormone-releasing hormone (LHRH) agonist, with biochemical response.

The patient kept a regular follow-up, with PSA levels consistently < 0.01 ng/dl. His only complaints were due to hormonal unbalance – minor depressive disorder, hot flashes, erectile dysfunction, and recurrent joint pain – but without new evidence of recurrent disease.

In an attempt to improve his symptoms, an intermittent therapy strategy was implemented maintaining close PSA monitoring.

At the time, the patient has 5 months of follow-up after LHRH agonist discontinuation, and no evidence of clinical, biochemical, or radiologic failure to date (approximately 4 years after pulmonary wedge resection).

## Case report 3

We present a 79-year-old, Caucasian male patient. He was a non-smoker retired army officer with a history of following multiple comorbidities: kidney stones, acute lithiasic pancreatitis, unstable angina (coronary artery disease with catheterisation/stent placement), hypertension, type-II diabetes mellitus, gastroesophageal reflux disease, and colonic diverticulosis.

The patient had undergone radical prostatectomy 17 years before and had a diagnosis of Gleason 7 prostate adenocarcinoma, pT_3a_ pN_x,_ with negative surgical margins. We did not have access to either pre- or post-operative PSA levels.

**Figure 3.**
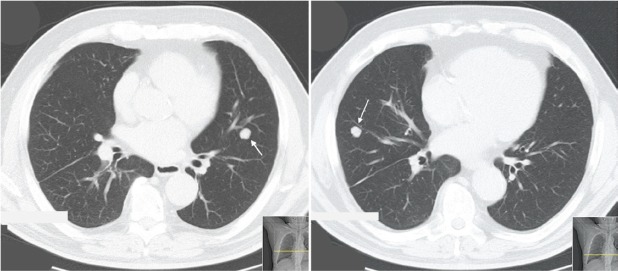
Thoracic CT scan (patient #3) exhibiting bilateral nodes. Arrow indicates lesion in the left lung (apical segment of the lower lobe) and right lung (anterior segment of the lower lobe).

Seventeen years later, he started complaining of insidious but progressive asthenia. Chest X-ray revealed two nodular opacities in the lower lobes of both lungs and thoracic CT scan presented multiple bilateral pulmonary nodules (middle right lobe, lingual, and apical segment of the left lower lobe) (Figure 3).

The assessment of disease extension (abdominal, pelvic, and bone scan) did not reveal metastasis in any other locations; PSA was 2.07 ng/dl. The first bronchoscopy did not show any direct or indirect changes to the respiratory tree (cytology and microbiology were non-pathologic).

**Figure 4.**
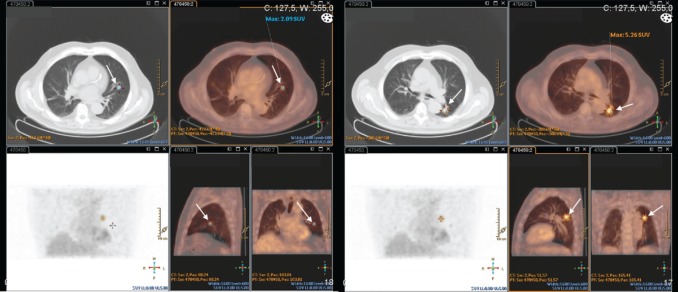
Patient #3 PET-CT-FDG: two hypermetabolic nodes in the lower lobe of the left lung – apical and anterobasal segments (arrows).

This exam was repeated one month later, and endobronchial lesions were found in the left lung. Biopsy confirmed metastatic adenocarcinoma of the prostate (PSA positive; TTF1, CK7, and CK20 negative). The PET–CT–FDG presented with the expression of two hypermetabolic secondary nodes in apical and anterobasal segments of the lower lobe of the left lung. Many other bilateral nodular lesions were present but without significant hypermetabolic uptake (Figure 4).

He initiated therapy with LHRH agonist along with bicalutamide (the latter only during the flare period), with PSA response. At the present time, he has five months of follow-up after initiating hormone therapy, with PSA levels < 0.01 ng/dl, and a recent chest CT revaluation showed a partial radiological response.

## Discussion

A PCa recurrence with pulmonary metastasis after radical prostatectomy in most cases means disseminated disease, with synchronous bone metastases. An isolated pulmonary metastasis is a very rare event. In autopsy series of patients with metastatic PCa, isolated pulmonary metastasis have been documented in less than 1% of cases [1, 2]. However, a few case reports on PCa patients with solitary lung metastasis have been published, proving that these cases indeed occur [3–8]. These three current reports demonstrate very unique and rare cases, with different management and outcomes.

In our second case, after wedge resection of the metachronous pulmonary metastasis and maintenance hormonal therapy with LHRH analogue, we managed to achieve a biochemical prolonged response (undetectable PSA level < 0.01 ng/ml).

Although surgical excision of PCa metastatic lesions is not generally advised, because the propensity for multiple other disease foci is probable to exist, it is possible that some patients might benefit from this treatment strategy.

In resemblance to what is done in other malignancies, the resection of a solitary pulmonary metastasis of a PCa recurrence after initial local therapy should probably be considered only in highly selected patients.

The finding of a solitary metastatic lesion in the setting of PCa does not necessarily mean that the patient has widely disseminated disease. In rare cases, resection of an apparent solitary metastasis might lead to biochemical remission, although it is still unknown whether such resection will prolong survival [9–11].

Although with smaller follow-up, the favourable PSA and radiological response in our second patient is promising, and in conformity with the conjecture that hormone naive patients with lung metastases may be good responders to androgen deprivation therapy.

## Conclusion

Isolated pulmonary metastases from PCa are extremely rare. We report one of the most numerous series. Surgical excision of solitary or oligometastatic lung secondary lesions, and hormonal therapy may play an important role in long-term disease control. It is considered necessary to assess more cases to understand the exact role of metastatic resection instead of hormonal treatment alone, but highly selected patients might be considered for the resection of pulmonary metastasis after primary localised treatment for PCa.

We should also emphasise the importance of considering PCa in the differential diagnosis of male patients presenting with pulmonary nodules, even in the absence of skeletal metastases.

## Disclosure

All the authors of this manuscript declare no conflicts of interest.
